# The role of audiovisual congruence in aesthetic appreciation of contemporary music and visual art

**DOI:** 10.1038/s41598-024-71399-y

**Published:** 2024-09-09

**Authors:** Lauren Fink, Hannah Fiehn, Melanie Wald-Fuhrmann

**Affiliations:** 1 Department of Music, Max Planck Institute for Empirical Aesthetics, Frankfurt Am Main, HE Germany; 2 Max Planck-NYU Center for Language, Music, & Emotion, Frankfurt Am Main, HE Germany; 3 Department of Psychology, Neuroscience & Behaviour, McMaster University, Hamilton, ON Canada; 4 Institute of Psychology, Goethe University, Frankfurt Am Main, HE Germany

**Keywords:** Multisensory integration, Digital art museum, Web-based data collection, Time spent, Feeling moved, Enjoyment, Human behaviour, Sensory processing, Perception

## Abstract

Does congruence between auditory and visual modalities affect aesthetic experience? While cross-modal correspondences between vision and hearing are well-documented, previous studies show conflicting results regarding whether audiovisual correspondence affects subjective aesthetic experience. Here, in collaboration with the Kentler International Drawing Space (NYC, USA), we depart from previous research by using music specifically composed to pair with visual art in the professionally-curated *Music as Image and Metaphor* exhibition. Our pre-registered online experiment consisted of 4 conditions: Audio, Visual, Audio-Visual-Intended (artist-intended pairing of art/music), and Audio-Visual-Random (random shuffling). Participants (N = 201) were presented with 16 pieces and could click to proceed to the next piece whenever they liked. We used time spent as an implicit index of aesthetic interest. Additionally, after each piece, participants were asked about their subjective experience (e.g., feeling moved). We found that participants spent significantly more time with Audio, followed by Audiovisual, followed by Visual pieces; however, they felt most moved in the Audiovisual (bi-modal) conditions. Ratings of audiovisual correspondence were significantly higher for the Audiovisual-Intended compared to Audiovisual-Random condition; interestingly, though, there were no significant differences between intended and random conditions on any other subjective rating scale, or for time spent. Collectively, these results call into question the relationship between cross-modal correspondence and aesthetic appreciation. Additionally, the results complicate the use of time spent as an implicit measure of aesthetic experience.

## Introduction

Multi-modal artforms, like dance, opera, and film, are highly-sought and can be deeply moving. These artforms involve tight connections between, at least, auditory and visual information. While much previous work has focussed on the stimulus features^1–5^, affective states^6,7^, developmental trajectories^8,9^, or semantic/cultural associations^10–12^ that drive cross-modal mappings (see^13–15^ for reviews), here we focus on whether correspondence between music and image has an influence on the aesthetic experience, and if so, how. We do not wish to enter the debate as to what specifically constitutes an aesthetic experience; it is a multifaceted phenomenon, with many contributing factors, such as person-level traits, contextual factors, cultural background, expectations, etc., with differing interpretations and underlying assumptions depending on whether one comes from a philosophical vs. psychological tradition^16^. We proceed with a similar working definition of aesthetic experience as that proposed by Wald-Fuhrmann et al.^16^ for music: “a person’s phenomenal state while attending to and internally interacting with a sequence of sounds primarily for the sake of its perceptual and formal properties and their possible meaning, but not so much its real-life information value.” In the current case, we examine participants’ responses to both sequences of sounds and visual images. Specifically, via a collaboration with the Kentler International Drawing Space (Brooklyn, NY, USA), we use a subset of the materials from their traveling exhibition, *Music as Image and Metaphor*, co-curated by David Houston and Florence Neal. At the time of writing, the exhibition has been presented at the Kentler in Brooklyn, New York, at the Bo Bartlett Center in Columbus, Georgia, at the Ohr-O’Keefe Museum of Art, in Biloxi, Mississippi, and online: https://www.kentlergallery.org/Detail/exhibitions/442. The exhibition consists of visual selections from the Kentler Flatfiles related to the theme of music, as well as one minute musical responses–composed specifically for these visuals–by Allen Otte (percussionist-composer) and Michael Kowalski (composer-pianist). Our research questions were generated with the curators and composers of the exhibition. Even though the composer-musicians had taken their task of musical responses very seriously, we wondered if it mattered which musical excerpt was paired with which piece, or, if the inclusion of music, in general, enhanced the visitors’ aesthetic experience. This question came from an observation of one of the curators: that visitors seemed to be spending more time in this exhibition, compared to previous exhibitions, perhaps because of the music.

There are only a few previous studies examining the effects of audiovisual correspondence on aesthetic evaluations. In one example, Siefke and Arielli^17^ studied how buildings (baroque vs. modern) are rated when they are presented with music (baroque vs. modern). They found that, in congruent presentations (where stimuli in both modes had the same style), the architecture was rated as more balanced, more coherent and more complete; in other words, congruence led to more positive aesthetic evaluations. In another example, Limbert and Pozella^18^ investigated how paintings and music are evaluated when they match, when they do not match, and when they are presented separately. In their study, the matched condition consisted of impressionist paintings and music chosen by the experimenters to match the impressionist paintings (2 min from “Le Jardin Féerique” by Maurice Ravel) or abstract paintings and atonal music chosen to match the abstract paintings (2 min excerpt from Anton Webern’s “Fünf Stücke für Orchester”). The non-matched condition consisted of impressionist paintings and atonal music and vice versa. Already one might note that these pairings are debatable: Ravel’s piece was composed much later than the impressionist paintings; it is almost from the same time as the Webern piece, which, in turn, is much older than some of the abstract paintings. Limbert and Pozella^18^ found more extreme ratings on Active–Passive and Ugly–Beautiful scales when music and painting style matched: Impressionistic paintings were rated as more beautiful and passive when paired with matching music. Abstract paintings were rated as uglier and more active when paired with matching music^18^. They concluded that listening to matching music while viewing the paintings *intensified* the aesthetic experience.

In another variation on the mapping between congruence and aesthetic experience, Fekete et al.^11^ recently found that pairing Klimt’s *Beethoven Frieze* with excerpts of Beethoven’s Ninth Symphony (its musical inspiration), led to longer time spent in the art museum, as compared to the painting with no music. However, contrary to their hypotheses, there were no differences in aesthetic enjoyment in their between-subjects design (comparing music + painting to painting-only). Similarly, the results of a study by Rančić and Marković^19^ suggest that the correspondence of music and art does not influence aesthetic experience. They examined if higher congruence between paintings and music induces a higher aesthetic liking of the painting. In their experiment, participants were presented with abstract paintings and jazz excerpts that matched more or less well in terms of regularity and complexity. These dimensions had been rated in a separate experiment by separate participants. Rančić and Marković^19^ found that participants rated congruent painting-music pairings as more highly matched than incongruent pairings, but this congruency in regularity and complexity had no effect on aesthetic liking. According to the authors, one possible explanation for the result could be the research question. Since the participants were only asked how much they liked the pictures (not the overall experience of pictures plus music), it would be possible for them to divide the aesthetic experience and ignore the music. However, it is questionable whether it is possible to clearly divide the aesthetic experience.

Given these previous studies, it seems more than plausible that there can exist crossmodal correspondence between complex art and music (see also^6,12^ and^13^ for review) but the effect of such correspondence on aesthetic experience remains unclear. One potential reason for conflicting findings is that “correspondence” has been operationalized differently in different studies; for example, that a painting and a piece of music come from the same period and/or have styles for which the same terms are used (e.g., abstract) does not necessarily mean that they share relevant properties. We thus sought to investigate the relationship between audiovisual correspondence and aesthetic experience further. Unlike all previously mentioned studies, which used pre-existing paintings and musical excerpts and paired them according to different characteristics (experimenter or participant-judged), here we use contemporary music composed and/or curated specifically to accompany pieces of contemporary visual art. That is, we use piece-specific, artist-intended pairings, all from a contemporary/avant garde style. All pieces were completely unfamiliar to participants, and likely the contemporary musical/artistic style was too. Additionally, unlike many previous studies, we used a completely balanced, within-subjects design, ensuring the ability to compare across modalities (audio-alone, visual-alone, intended audiovisual pairings, and shuffled audiovisual pairings) and precluding the possibility for pre-existing group-level differences. Because the curators of the *Music as Image and Metaphor* exhibition had noticed people spending more time in the gallery than normal, we thought it fitting to use “time spent” as our main behavioral marker of aesthetic interest, in addition to subjective self-reports.

In the auditory domain, time spent listening has been used as a measure of attention, related to exploratory behavior, or perceptual curiosity^20^, analogous to looking time in the visual domain^21,22^. A recent series of experiments in the music domain affirms that listening times are related to stimulus novelty / complexity, as well as a persons’ subjective enjoyment^23^. Janata et al.^23^ unite many factors identified in prior studies, showing that: ‘mood congruency,’ ‘enjoyment,’ and ‘interestingness of the stimuli’ all have direct effects on listening time, while ‘complexity’ has an indirect effect through ‘interestingness.’ Other measured variables, like ‘groove’ and ‘familiarity’, also have indirect effects, but mediated through ‘enjoyment.’ Thus, in the current study, in addition to time spent, we measured all of its proposed direct mediators: *mood congruency, enjoyment,* and *interestingness*, via self-report. We also added an additional measure asking participants how *‘moved’* they felt by the piece.

Unlike the experience in the physical or online gallery, which consisted of visual art, paired with music that could be controlled by the visitor, in our experiment, we wanted to control the onset of stimulus presentation and also to compare uni- vs. multi-modal conditions in both directions. Thus, our design consisted of 4 conditions: Audio, Visual, Audio-Visual-I (artist-intended pairing of musical piece with visual piece), and Audio-Visual-R (random pairing of audio and visual from within the stimulus set). Thus, in contrast to previous studies, here we have a piece-specific artist-defined ‘congruent’ condition, which we think is important, given previous studies that have operationalized correspondence mostly on the group level by similar artistic styles^17,18^, semantic associations^11^, or lay participant ratings^19^ (see^13^ for review). Our random condition is similarly not as incongruent as previous studies which often contrasted completely different artistic styles^17,18^ and/or levels of complexity^19^. Here, we use a balanced shuffling of stimulus combinations (Latin Square design), such that, across participants, e.g., the same audio occurs alone, with its intended visual pairing, and with a random visual pairing from the exhibition. In this way, we ensure that stimulus is not confounded with modality. Additionally, our design allows us to study effects within, rather than between participants. The time between stimulus onset and when participants clicked ‘Next’ was measured for each trial. After clicking next, participants were prompted to answer questions about their enjoyment, feeling moved, how interesting they found the piece, how well the piece matched their mood, and, in audiovisual trials, the degree to which they felt there was a correspondence between the audio and visual sensations they were experiencing.

We hypothesized that the least time would be spent on visual images alone, followed by audio alone, then AV-R, then AV-I. As has been shown in previous studies, we hypothesized that time spent would be positively correlated with self-reported subjective experiences. All study hypotheses, procedures, and analyses were pre-registered (^24^ https://osf.io/hjgc5/). Readers can view a demonstration of the experiment, from the participants’ perspective, by following this link: https://www.labvanced.com/player.html?id=33023.

## Results

**Fig. 1:**
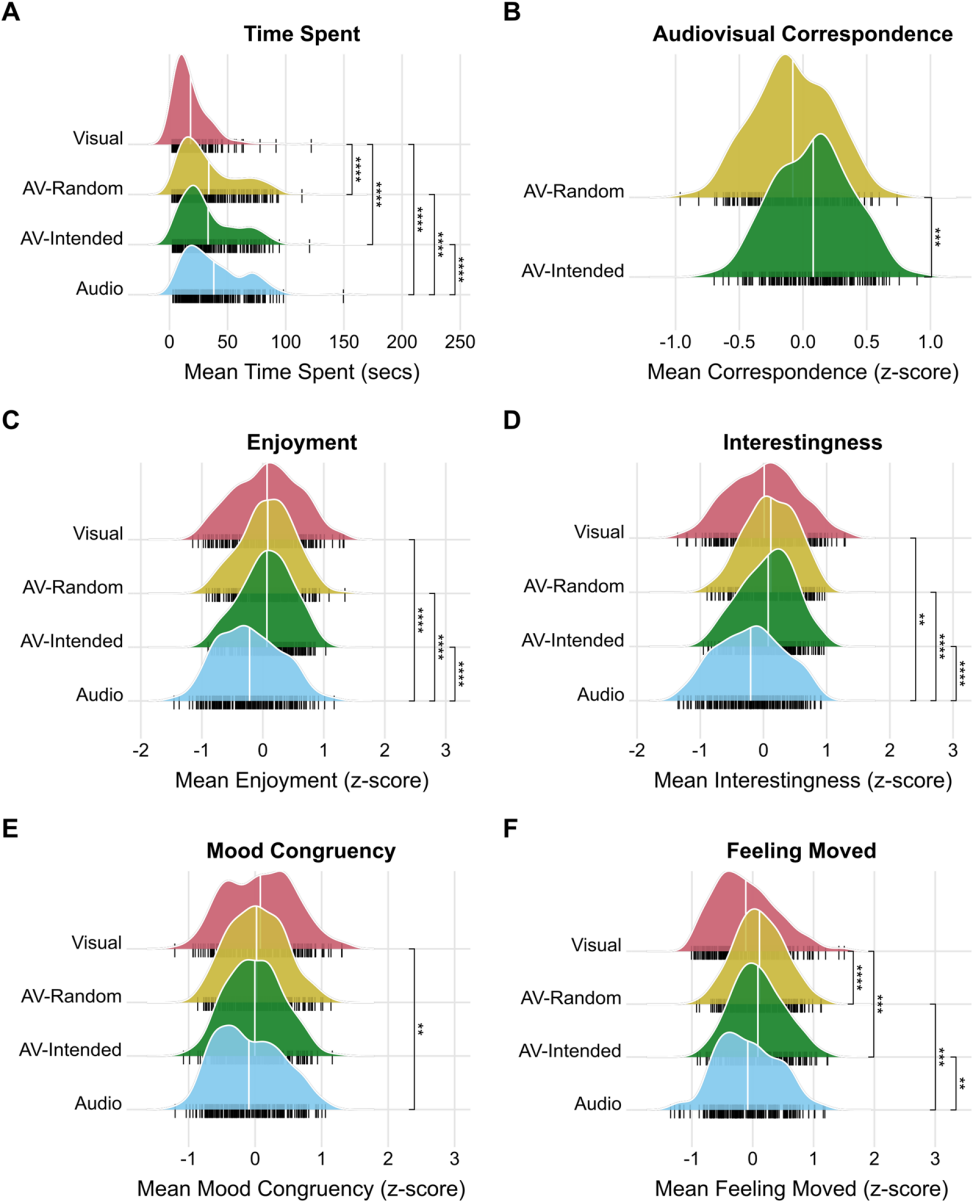
Comparison of all dependent measures in each condition. In all subplots, probability density functions show the shape of the data distributions by condition, with the median indicated via vertical white line. Small black vertical lines under each density plot represent individual participants (N = 201). For all dependent variables, a main effect of condition was significant. Lines, stars, and ‘ns’ represent the significance level of planned contrasts. * *p* < = 0.05; ** *p* < = 0.01; *** *p* < = 0.001 ; **** *p* < = 0.0001. (**A**) Time Spent (in seconds) was measured by calculating the time lapsed between onset of the stimulus and participants’ clicking the ‘next’ button. (**B**) Audiovisual correspondence represents participants’ rating of the degree to which they felt there was a correspondence between the audio and visual sensations they were experiencing. (**C–F**): Subjective ratings of enjoyment, interestingness, mood congruency, and feeling moved, respectively.

As outlined in our pre-registration^24^, we planned to conduct ANOVAs to understand the effect of modality on each dependent measure (time spent, and all five subjective ratings). We will start with the implicit time spent measure (i.e., the time participants spent with each piece [each trial] before clicking the ‘next’ button). We found that, across the experiment, participants spent most time, on average, in the Audio condition (mean: 37.08 secs, SD: 25.60 secs), followed by AV-R (mean: 32.64, SD: 24.42), AV-I (mean: 31.68, SD: 23.28), and V (mean: 16.73, SD: 15.36); see Fig. 1A. A one-way repeated measures ANOVA confirmed a significant main effect of condition, F (1.95, 390.22) = 102.44, *p* < 0.001. T-tests comparing conditions to each other were all significant (< 0.001), except that there was no significant difference between the random vs. intended audiovisual pieces (AV-R vs. AV-I conditions; *p* = 0.279). This pattern of results remained identical whether we used raw time spent (in seconds) or transformed to log time spent, as some previous studies have done (e.g., Janata et al., 2018; Brieber et al., 2014); see additional analyses in accompanying R Notebook.

Despite the lack of difference between AV-I and AV-R conditions in terms of *Time Spent*, participants’ self-reported ratings regarding the degree to which they felt *Correspondence* between the audio and visual sensations they were experiencing were significantly higher in the AV-I, compared to AV-R condition (Welch’s two sample t-test: *t *(400) = 5.27, *p* < 0.001; see Fig. 1B). Nonetheless, AV-I vs. AV-R conditions showed no significant differences on any other subjective scales, see Fig. 1C–F. We found a significant main effect of condition on *Enjoyment*, F (2.76, 552.98) = 13.83, *p* < 0.001, with contrasts showing that AV-I and AV-R are higher in enjoyment than A, but not V, or each other; V was more enjoyable than A (all *p* < 0.001; Fig. 1C). We also found a main effect of condition on *Interestingness*, F (2.65, 526.68) = 13.06, *p* < 0.001. Similarly, contrasts showed that anything including visuals was more interesting than audio alone (see Fig. 1D). There were no significant differences between any other conditions. With respect to *Mood Congruency*, follow-up comparisons to the main effect of condition on mood congruency ratings, F (2.84, 564.37) = 3.61, *p* = 0.015, showed only one significant contrast, with the Visual condition being higher in mood congruency than the Audio condition (t = 2.83, *p* = 0.031; Fig. 1E). Regarding feelings of being *Moved*, there was again a main effect of condition, F (2.83, 561.24) = 9.12, *p* < 0.001. Contrasts showed that anything bi-modal (AV-I and AV-R) was significantly more moving than anything uni-modal (A or V), all *p* < 0.05; see Fig. 1F. There were no significant differences within uni-modal (A vs. V) or bi-modal (AV-I vs. AV-R) conditions, all *p* = 1.

Feeling more moved in bi-modal conditions, is in line with participants’ self-reported preferences at the end of the experiment. When asked whether they had a preference for audio, visual, or audiovisual material, 62% of participants preferred audiovisual, while 15% preferred visual, 12% preferred audio, and 11% had no preference. Overall, participants reported enjoying the experiment: on a scale from 0 to 100, the average overall enjoyment rating was 85 (+ /− 20). In terms of how likely participants reported they were to go see a similar exhibition in the real-world, the average rating was 55 (+ /− 32).

## Discussion

Our results point to a highly interesting disconnect between audiovisual correspondence and aesthetic evaluation. Participants rated stimuli in the AV-Intended condition higher in correspondence than those in the AV-Random condition. However, the AV-I and AV-R conditions showed no difference with respect to any other subjective ratings or time spent. In other words, artist-intended AV pairings were rated more highly on the correspondence scale but this higher correspondence had no bearing on aesthetic experience. These results are in line with previous studies showing correspondences between art and music^13,25^, and contribute to the debate around whether such correspondences affect aesthetic experience^11,17–19^.

With respect to time spent in our four different experimental conditions, we find that the inclusion of music increases viewing times of visual art, compared to visual art alone. However, participants actually spent the most time when only audio was present. While these results replicate previous work showing increased time spent when music is paired with art^11^, they also call into question the suitability of *Time Spent* as an implicit index of aesthetic experience. Our balanced experimental design allowed us to determine that, though participants spent more time in audiovisual conditions, compared to the visual-only one, they actually spent the most time in the audio-only condition. There is of course the obvious explanation that audio evolves over time and therefore requires more time to be properly evaluated, whereas a gestalt of the visual scene can be obtained relatively quickly. However, previous studies in the visual-only domain have shown positive relationships between time spent and aesthetic evaluations^26^. Similarly, previous studies with audio-only have shown relationships between aesthetic appreciation and time spent^23^. Further, in previous audiovisual studies, time spent was again associated with experienced chills and pleasantness of the painting^11^, though the authors note that the increased time spent could also have been due to the duration of the audio. So, why in our study did people feel most moved in audiovisual conditions but spend most time in the audio condition? Perhaps it could also be argued that in the current context, participants spent only as much time as they needed to fulfill subsequent task demands (subjective ratings), and did not indulge in watching or listening as long as they liked, but only how long was necessary to form an opinion. Nonetheless, our exploratory results (see accompanying R notebook) suggest that time spent is in fact weakly correlated with feeling moved across the whole experiment, but that this effect is almost exclusively driven by the audio and AV-R conditions. One might then speculate that the greater the ambiguity, the more strongly associated time spent becomes with aesthetic experience. However, future work must explore such a notion further. Additionally, it is important to bear in mind in future studies that using time spent as a dependent measure may require particular care in terms of both task design and comparisons across modalities.

Our participants found bi-modal conditions to be more moving than unimodal ones, and spent more time in all conditions involving audio, compared to visual only. These results speak to the potential relevance of music inside art galleries. Here, we found that participants spent, on average, 32 s in audiovisual conditions, whereas previous art gallery studies have found that participants spend an average of only 28.6 s with a painting^21^ (our results suggest an average of only 17 secs for visual-only conditions). Further, given recent work showing that engaging with online visual art can enhance well-being^27^, the current study would go one step further to suggest that online art-based interventions could incorporate music to be even more impactful. While it was not measured in the current study, self-relevance of the art/music might be another important variable to consider in future studies^28^.

While one interpretation of the current findings regarding a lack of difference between intended vs. random audiovisual pairings is that *any* audio can be used to enhance the visual experience, we would like to argue that perhaps the pairing did not matter in the current context because all audio stimuli used here were of a similar avante-garde style, within the context of the *Music as Image and Metaphor* exhibition. Such thinking is in line with previous work regarding structural and/or aesthetic similarities between artforms (see^13^ for review). In other words, though we shuffled which music was paired with which piece of visual art in the AV-R condition, these random pairings were still from the exhibition and therefore of a similar contemporary style. We have to imagine that, had the audio in the AV-R condition been completely incongruent with the visual aesthetic (e.g., music not from this exhibition, but rather from a different contemporary composer, or even different musical style, such as country music, or period, such as baroque), the results might change. Indeed, a recent study by Braun et al.^29^ showed that different types of background music influenced museum visitors’ experience of a Kandinsky painting. Specifically, the intended emotional valence of the music (happy, sad, peaceful, scary) predicted participants’ pleasantness ratings of the painting. Additionally, participants’ liking of the music influenced their liking of the painting. Similarly, many decades ago, Lindner and Hynan^5^ showed that participant-rated semantic polarity associations (e.g., ordered–chaotic) changed when pairing the same paintings with either minimalist or avante-garde music. In both of these previous studies, there were no AV correspondence ratings so a direct comparison with the present study is not possible, but future work might explore whether AV pairings matched in rated congruence, but differing in valence, affect aesthetic experience. Indeed, much previous literature has focused on the role of emotion in mediating cross-modal correspondences^6,7,12,14^.

In summary, the current study exemplifies the meaningful and interesting scientific questions that can be answered through collaboration with artists, musician-composers, and museum curators. Such collaborations serve multiple purposes. First, they increase the visibility and reach of artistic material, and conversely the reach of the associated scientific output. To elaborate, online participants actually went out of their way to contact the experimenters to say how much they enjoyed the experiment and ask for suggestions about where to find similar musical material. Such feedback from participants was deeply meaningful to the authors of this study, and also increased the reach of the visual artists and composer-musicians involved. Conversely, via a panel discussion that took place at the Kentler International Drawing Space in Brooklyn, NY, USA, during the final weeks of this exhibition, Fink had the opportunity to share the preliminary results of the current study with a public, non-scientific audience, and to engage in a public dialogue with the exhibition curators and composers (recording available: https://www.kentlergallery.org/Detail/events/540). This type of outreach increases the visibility of the arts in STEM (STEAM) and exposes the public to the scientific process. Second, such collaborations allow for unexpected and exciting contributions to the scientific literature. Given that artistic and scientific innovations can bi-directionally influence one another^30,31^, the approach of taking creative exhibitions as inspiration for scientific work may be a particularly fruitful one (see e.g.,^32^, who transgress the boundaries of real-world, interactive cognitive neuroscience experiments, and performance art, taking Abramović’s *The Artist is Present* as inspiration). We look forward to the continued collaboration, and blurring boundaries, between the arts and sciences, which promises to bring not only new scientific insights, but also deeply meaningful aesthetic experiences for participants.

## Methods

Study hypotheses, procedures, and analyses were all pre-registered (^24^; https://osf.io/hjgc5/). The most pertinent details are repeated below. Readers can participate in the study online at: https://www.labvanced.com/player.html?id=33023.

### Stimuli

Overall we had access to 40 paintings and 40 corresponding music pieces, of which 20 pieces were composed by Allen Otte and 20 by Michel Kowalski. Due to previous studies showing that abstract and representational art elicit different psychological states^15,33^ and viewing patterns^34^, we used only abstract paintings (and their corresponding music pieces). Therefore, we excluded 17 paintings in which objects could be identified. Of the remaining 23 pieces we randomly selected 12, counterbalanced for both music composers. Though Kowalski and Otte responded musically to the visual works in a variety of “modes”: as a gestural dialogue, a thematic extension or development, a compositional analogy, a soundtrack, or a spontaneous reaction, in the current study, we do not explore the subspace of the different types of responses, but we have balanced the number of compositions by the two composers.

The stimuli were presented in four different conditions: Audio (A), Visual (V), Audio-Visual Random (AV-R), and Audio-Visual Intended (AV-I). We created three different stimulus groups via a Latin square design. Thereby, each piece was presented only once to each participant, but was presented in each condition across participants. In total, 16 stimuli were presented to each participant. Participants were randomly assigned to a stimulus group and, within each run, the presentation of the stimuli was randomized. A list of stimuli for one of the groups is displayed in Table 1. All stimuli can be viewed/listened to in the Kentler International Drawing Space’s digital exhibition gallery: https://www.kentlergallery.org/Detail/exhibitions/442.

**Table 1:** One possible set of stimuli participants might experience.

Condition	Audio	Visual
Audio	Allen Otte, *Excerpt from Qu Xiao Song, Lam Mot, 1991 performed by Percussion Group Cincinnati.* Link	N/A
	Allen Otte, *Excerpt from Vaster Than Empires, improvised performance, 2017, Erica Dicker, amplified violin, Paul Schuette, computer and synthesizer.* Link	N/A
	Michael Kowalski, *Remix of orchestral parts from A Ascensão e a Queda do Primeiro Mundo, 2017, with percussion, 2020.* Link	N/A
	Michael Kowalski, *Reduction and recombination of L.v. Beethoven, Eleven Bagatelles, Op. 119, with percussion, 2020*. Link	N/A
Visual	N/A	Hannah Israel, *The same and other* (2019). Link
	N/A	Ralph Kiggell, *Progression (2011).* Link
	N/A	Scott Pfaffman, *Note* (2016). Link
	N/A	Richard Howe, *010721/6* (2001). Link
AV-I	Allen Otte, *Excerpt from Mark Saya, The Simurgh, 1993; prepared piano, 2020.* Link	Abbie Goldstein, *Untitled 1* (2019). Link
	Allen Otte, *Mark Saya, Chopin A major Prelude Revisited, 1983, performed by Percussion Group Cincinnati.* Link	Jim Napierala, *Harmonic* (2007). Link
	Michael Kowalski, *Remix of Hotsy-totsy for analog synthesizers, 1977, with percussion, 2020.* Link	Kazuhiro Nishijima, *Untitled* (2002). Link
	Michael Kowalski, *Remix, 2023, of excerpt from memoriam: Sydney Toler for analog synthesizers and sampled sound, 1973.* Link	Jiří Kornatovský, *Basic Story* (1990). Link
AV-R	Allen Otte, *Iberian adulfe (medieval frame drum), 2020.* Link	Margaret Neill, *Respite 3* (2013). Link
	Allen Otte, *Fire bell, cymbal, Javanese Gender, 2020.* Link	Florence Neal, *Trio II* (2006). Link
	Michael Kowalski, *Remix, 2020, of Variations for String Sextet, 1973, with percussion, premiered at the University of Iowa, conducted by Gerald Chenoweth.* Link	Gahae Park, *Music Drawing in Blue* (2001). Link
	Michael Kowalski, *Percussion mix, 2020, with excerpts from Fakebook for piano solo, 1976.* Link	Mary Judge, *Automatic Writing Series no. 17* (1999). Link

Participants were assigned to one of three groups, each of which was a Latin square reshuffling of the stimuli, such that, across participants, each stimulus was presented in each condition. Links are directly to the Kentler exhibition website. Note that, for audio, the links do not exist on the exhibition website independent of their intended visual pairings. To experience the stimuli as participants did, please visit our experiment demo.

### Procedure

All experimental procedures were approved by the Ethics Council of the Max Planck Society, and undertaken with written informed consent of each participant. All research was performed in accordance with the Declaration of Helsinki. Data were collected online from participants’ own home. We used LabVanced^35^ for stimulus presentation and data recording and Prolific (www.prolific.co) for participant recruitment.

Participants were told that they would be presented with 16 pieces that they could view/listen to as long as they wanted. They were aware that clicking “next” would end the presentation and that they could take a break between each piece. They were told that the pieces they would experience would sometimes contain both music and an image, sometimes only one or the other. We explained that the questions after each presentation referred to the *whole* experience, not to single components (just the painting or just the music, in the case of audiovisual presentation).

After experiencing each piece, participants were asked to answer a series of questions on a sliding scale, ranging from 0 to 100 (with a number in the middle indicating their current selection). The slider always started at 0 and was marked at the end points with labels for 0 (not at all) and 100 (completely). The questions asked were (1) “The piece matched my current mood,” (2) “The piece moved me,” (3) “I enjoyed the piece,” and (4) “I found the piece interesting.” After the presentation of an audiovisual piece, they were also asked “To what degree did you feel there was a correspondence between the audio and visual sensations you were experiencing?”.

After all stimuli had been presented, we asked participants about their proneness to aesthetic experience, measured via the Aesthetic Responsiveness Assessment (AReA^36^), and their musicianship^37^. Other person-level variables collected were participants’ overall enjoyment of the study, which condition they felt they preferred (A, V, AV or none), the probability that they would go to see/hear similar pieces in a museum/concert, and basic demographic information (gender, age, country).

### Participants

Participants were compensated £11/hr for their time. No participants had previously seen the Kentler exhibition. Two-hundred seven participants took part in the study. Six participants were removed from the dataset because they did not complete the task and/or they did not have normal, or corrected-to-normal hearing and vision, resulting in a final sample of 201 participants. Responses to the AReA and demographic questionnaires are missing for 6 of these 201 participants. Rather than excluding these participants on the basis of missing person-level data, we include them in our analyses for the main experimental task. Demographic information, including self-reported musicianship, and scores on the AReA assessment^36^ are reported below in Table 2. Participants came from 23 unique countries (top three: United Kingdom, Portugal, Poland); all were fluent in English.

**Table 2:** Sample characteristics.

Age (years)	Gender	Musicianship	AA	IAE	CB	AReA
31 (12) Min: 18 Max: 68	Women: 73 Men: 117 Non-binary: 4 Prefer not to say: 1	2.2 (0.95) Min: 1 Max: 5	3.4 (0.69) Min: 1.1 Max: 5	2.5 (0.92) Min: 1 Max: 5	2.0 (0.92) Min: 1 Max: 5	2.6 (0.74) Min: 1.1 Max: 4.8

Musicianship represents self-reported musicianship on the Ollen scale (ranging from 1 to 6: non-musician to professional musician).

*AA* aesthetic appreciation*, IAE* intense aesthetic experience, *CB* creative behavior, AReA represents the overall Aesthetic Responsiveness score, an average of the three subscales (AA, IAE, CB).

### Planned analyses

The data are publicly accessible on GitHub (https://github.com/lkfink/Kentler_MIM_Behavior), as is the code to recreate all analyses and figures. All analyses were conducted in open-source language R and the RStudio environment^38^.

### Condition-averaged analyses

Our main interest was in comparing differences in participants’ subjective ratings and time spent between conditions. The time participants spent with the pieces was defined as the time from the onset of the stimulus until the time the participant clicked on the “Next” button. The other dependent variables were subjectively rated by the participants on a scale from 0 to 100 (see Procedure). These variables were measured for every trial. Within participant and scale, we z-score normalized ratings data. Time spent data were not normalized and are reported in seconds. For each individual we calculate the mean by condition. For all variables of interest, planned one-way repeated measures ANOVAs were conducted with condition as the within-subjects factor and subjective rating (or time spent) as the dependent variable. Post-hoc contrasts to compare individual conditions were Holm-corrected for multiple comparisons.

### Person-level analyses

The person-level variables which were measured after the presentation of all stimuli (aesthetic experience, musicianship, overall enjoyment, preference, probability of seeing similar pieces again, and basic demographic information) were used for exploratory purposes and for the planned mediation analysis. The AReA scale was scored according to the scoring rubric from the authors of the original instrument^36^, resulting in three scores for (1) aesthetic appreciation (AE), (2) intensity of aesthetic experience (IAE), and (3) creative behaviours (SB), as well as an overall composite score (see Table 2).

### Mediation analysis

Our pre-registered mediation analysis no longer made sense once time spent was shown to vary by condition and be unrelated to aesthetic preferences. Additional exploratory analyses related to *Time Spent* are included in the accompanying R notebook.

## Data Availability

All code and data required to reproduce the analyses reported in this manuscript are available on GitHub: https://github.com/lkfink/Kentler_MIM_Behavior and OSF: https://osf.io/hjgc5/.
